# Protective effects of isoflavones on alcoholic liver diseases: Computational approaches to investigate the inhibition of ALDH2 with isoflavone analogues

**DOI:** 10.3389/fmolb.2023.1147301

**Published:** 2023-02-27

**Authors:** Wook Lee, Seung-Jin Kim

**Affiliations:** ^1^ Department of Biochemistry, College of Natural Sciences, Kangwon National University, Chuncheon, Republic of Korea; ^2^ Kangwon Institute of Inclusive Technology, Kangwon National University, Chuncheon, Republic of Korea; ^3^ Global/Gangwon Innovative Biologics-Regional Leading Research Center (GIB-RLRC), Kangwon National University, Chuncheon, Republic of Korea

**Keywords:** alcoholic liver disease (ALD), Aldehyde dehydrogenase (ALDH2), isoflavones, computer simulatioin, chemical structure

## Abstract

Excessive and chronic alcohol intake can lead to the progression of alcoholic liver disease (ALD), which is a major cause of morbidity and mortality worldwide. ALD encompasses a pathophysiological spectrum such as simple steatosis, alcoholic steatohepatitis (ASH), fibrosis, alcoholic cirrhosis, and hepatocellular carcinoma (HCC). Aldehyde dehydrogenase (ALDH2) is the most vital enzyme that produces acetate from acetaldehyde and is expressed at high levels in the liver, kidneys, muscles, and heart. The ALDH2*2 allele is found in up to 40% of East Asian populations, and has a significant impact on alcohol metabolism. Interestingly, several studies have shown that individuals with ALDH2 deficiency are more susceptible to liver inflammation after drinking alcohol. Furthermore, there is growing evidence of an association between ALDH2 deficiency and the development of cancers in the liver, stomach, colon, and lung. Isoflavone analogues are low molecular-weight compounds derived from plants, similar in structure and activity to estrogen in mammals, known as phytoestrogens. Recent studies have reported that isoflavone analogues have beneficial effects on the progression of ALD. This mini-review summarizes the current knowledge about the roles of isoflavone analogues in ALD and discusses the therapeutic potential of isoflavone analogues in liver pathophysiology. In particular, we highlight the significance of computational approaches in this field.

## 1 Introduction

The liver is one of the most important organs in the human body and is responsible for multiple pathophysiological functions, including digestion, synthesis, hormone metabolism, detoxification, and immune responses (Kalra et al., 2018). In particular, the liver plays a pivotal role in metabolism, which properly converts ingested food into nutrients and distributes them to various tissues (Dardevet et al., 2006). Liver diseases are caused by various harmful factors such as obesity, drugs, viruses, and alcohol. For example, when the body gains weight, hepatocytes are unable to break down lipids, which can lead to fatty liver disease (Parker et al., 2018; Kim et al., 2019; Geng et al., 2021). Acetaminophen and viral hepatitis are causes of acute liver failure (Larson et al., 2005; Manka et al., 2016). Excessive and chronic alcohol consumption leads to immune cell infiltration, and increased oxidative stress can cause alcoholic liver disease (ALD) (Parker et al., 2018; Parker et al., 2019; Kim et al., 2021; Yang et al., 2022). Genetic polymorphisms in enzymes involved in alcohol metabolism such as ALDH2 and cytochrome P450 2E1 (CYP2E1), are considered risk factors for ALD (Enomoto et al., 1991; Zeng et al., 2013; Wang et al., 2021). Mitochondrial ALDH (ALDH2) is a major enzyme involved in ethanol metabolism and detoxification of alcohol-derived acetaldehyde (Zakhari and Li, 2007). In addition, several studies indicate that ALDH2 participates in human pathophysiology including alcohol addiction (Pautassi et al., 2010; Zhang and Ren, 2011). Deletion of ALDH2 resulted in the acceleration of alcohol-induced liver inflammation but increase resistance to alcohol-induced steatosis and serum alanine aminotransferase (ALT) levels in mice (Kwon et al., 2014). Furthermore, ALDH2 deficiency significantly increases the progression of alcohol-associated liver cancer (Seo et al., 2019). Although the incidence of ALD is on the rise worldwide, there are limited treatment strategies or efficient drugs owing to the characteristics of irreversible liver diseases. Phytoestrogens can be classified into several subgroups, including isoflavones, flavonoids, coumestans, lignans and stilbenes (Dixon, 2004). Isoflavone analogues (daidzein, genistein, biochanin A, formononetin, glycitein and puerarin) have protective effects against ALD induced by various factors such as anti-inflammatory, antioxidant, anti-fibrotic, and anti-apoptotic signals (Alipour and Karimi-Sales, 2020; Kim, 2021). Thus, in this mini-review, we summarize recent advances in our understanding of the roles of isoflavone analogues in the development of ALD and address the results of computer simulations at the atomistic level regarding the interactions between isoflavone analogues and ALDH2.

## 2 Isoflavone analogues and ALDH2 in the progression of ALD

**FIGURE 1:**
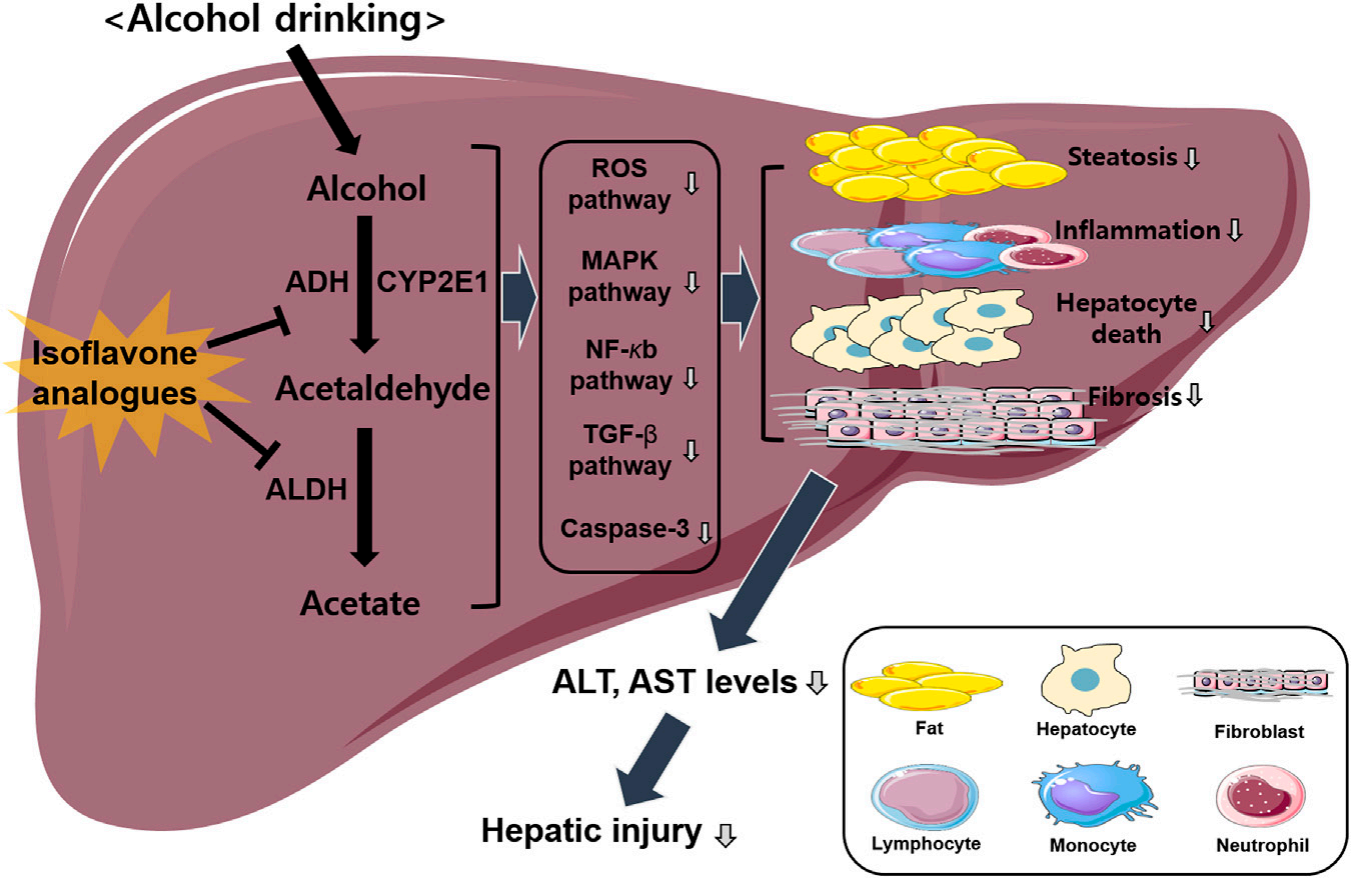
Overview of isoflavone analogues-induced protective effects against ALD. Alcohol drinking induces hepatic injury *via* activating several different pathways including ROS, MAPK, NF-κb, and TGF-β signaling. Isoflavone analogues have protective effects against ALD induced by various factors including inflammation, steatosis, fibrosis, and apoptosis. Finally, this pathway decreases hepatic injury *via* the inhibition of alcohol metabolism.

As mentioned in the previous section, isoflavone analogues have protective effects against ALD induced by various factors including inflammation, oxidation, fibrosis and apoptosis (Alipour and Karimi-Sales, 2020; Kim, 2021). ALDH2 is the most crucial alcohol metabolizing enzyme that produces acetate from acetaldehyde and is considered a risk factor for ALD (Zakhari and Li, 2007). Several studies have indicated that isoflavone analogues are promising therapeutic candidates that inhibit ALDH2 activity (Kimura et al., 2019; Alipour and Karimi-Sales, 2020; Kim, 2021; Zhang et al., 2022). Therefore, this section discusses the detailed mechanisms of isoflavone analogues in the progression of ALD. In particular, it will focus mainly on liver cells, including hepatocytes, macrophages, and other types of immune cells that mediate cross-talk between isoflavone analogues and ALDH2 in ALD (Figure 1).

### 2.1 Hepatocytes

Hepatocytes are the most abundant cells in the liver and play key roles in protein synthesis, cholesterol synthesis and detoxification (Zhou et al., 2016). The function of isoflavone analogues during ALD causes an alteration in the hepatocytes *via* changes in the expression of various genes (Alipour and Karimi-Sales, 2020); however, the role of isoflavone analogues in hepatocytes remains unclear. In 2016, Zhao et al. demonstrated that isoflavone analogues could inhibit chronic alcohol-induced hepatocellular apoptosis by inhibiting caspase-3 activity (Zhao et al., 2016). They selected genistein, which is one of the major isoflavones that is a potent inhibitor of ALDH2. Consequently, genistein treatment ameliorated chronic alcohol-induced liver injury, histopathological changes, and lipid peroxidation *via* inhibition of inflammatory signals such as nuclear factor-κB (NF-κB), monocyte chemoattractant protein-1 (MCP-1), interleukin-6 (IL-6), tumor necrosis factor-α (TNF-α), and transforming growth factor-β1 (TGF-β1) (Zhao et al., 2016). Similarly, Xie et al. demonstrated that genistein had a beneficial role against acute-on-chronic liver failure, which was reflected by decreased aspartate aminotransferase (AST) levels in serum and mitochondrial injury in hepatocytes (Xie et al., 2019). In addition, Zhao et al. reported that puerarin treatment clearly rescued alcohol-induced lipid degeneration in hepatic cells, cellular swelling, and focal necrosis *via* inhibition of oxidative stress in rats (Zhao et al., 2016). Li et al. indicated that puerarin had a protective effect against alcohol-induced liver injury by inhibiting immunotoxicity in hepatocytes through the regulation of the glycogen synthase kinase-3 beta (GSK-3β)/NF-κB signaling pathways (Li et al., 2013). Collectively, these observations indicate that isoflavone analogues exert beneficial effects on alcohol-induced liver injury *via* their anti-inflammatory and anti-apoptotic properties in hepatocytes.

### 2.2 Macrophages and other types of immune cells

Macrophages are large phagocytes found in all tissues in the body that play a crucial role in innate and adaptive immunity by recruiting other immune cells (Hirayama et al., 2017). Therefore, infiltration of macrophages or activation of resident Kupffer cells is an important phenotypic marker of ALD progression (Kim et al., 2021). A recent study reported that isoflavone analogues inhibited Kupffer cell activation and endotoxin receptor expression in a mouse model of chronic alcoholic liver injury (Zhao et al., 2016). Similarly, Tan et al. found that isoflavone analogues inhibit lipopolysaccharide (LPS)-induced inflammation in RAW264.7 macrophages *via* the MAPK and NF-κB signaling pathways (Tan et al., 2022). In addition, it has been reported that daidzein, an isoflavone analogues that is a potent and selective inhibitor of ALDH2, alters the expression of pro-inflammatory cytokines in macrophages and adipocytes (Sakamoto et al., 2016). Neutrophils are the largest white blood cells in mammals and play an important role in innate immunity (Rosales, 2018). Neutrophil infiltration into the liver is a major feature of early-stage ALD progression (Ramaiah and Jaeschke, 2007). Only a few studies have evaluated the relationship between isoflavone analogues and neutrophils in ALD. In 1998, Sadowska-Krowicka et al. reported that genistein treatment significantly decreased trinitrobenzenesulfonic acid (TNBS)-induced myeloperoxidase (MPO) activity, which is an index of neutrophil infiltration in a male Hartley guinea pig model (Sadowska-Krowicka et al., 1998). Hepatic stellate cells (HSCs) are an important component of the liver fibrogenesis involved in ALD (Zhang et al., 2016). Interestingly, Zhang et al. demonstrated that puerarin had a regulatory effect on alcoholic liver injury in rats by decreasing the levels of TGF-β and α-smooth muscle actin (α-SMA), which are key factors involved in liver fibrogenesis (Li et al., 2019). Consequently, isoflavone analogues may be useful in attenuating alcohol-induced liver damage through the regulation of macrophages and other types of immune cells in the liver.

## 3 Isoflavone analogues and ALDH2: Computational approaches

As discussed in the previous section, inhibition of ALDH2 activity can lead to the suppression of alcohol-induced liver diseases, and isoflavone analogues have gained attention as promising drug candidates to inhibit ALDH2 activity. So far, several studies have been carried out to aid the understanding of the interactions between ALDH2 and isoflavone analogues. In this section, we will mainly focus on studies using computer simulations to examine the molecular basis for these interactions and will discuss the capability of computer simulations in that regard.

**FIGURE 2:**
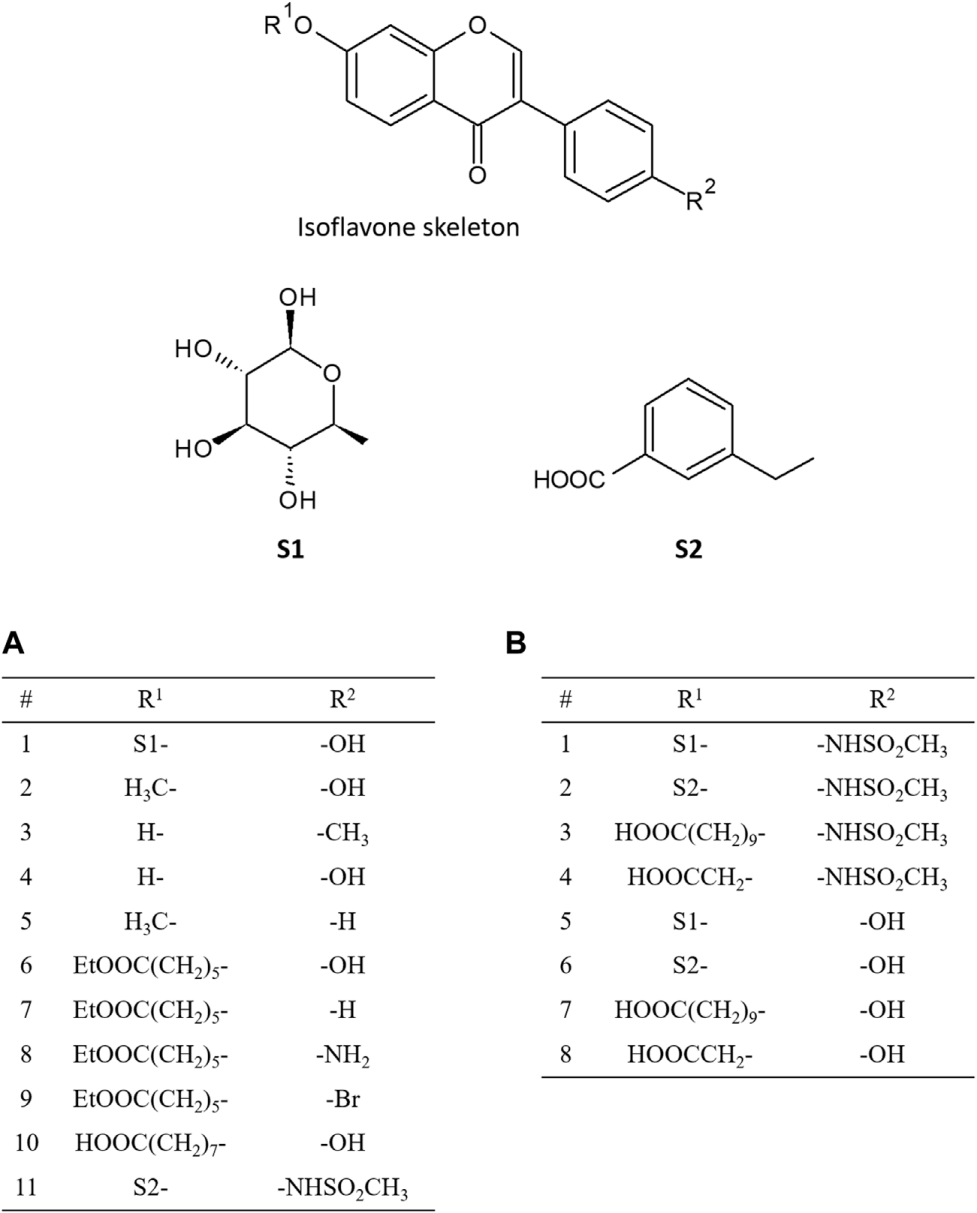
Chemical structures of isoflavone analogues. The substituted groups of daidzin at R^1^ and R^2^ position are S1- and -OH, respectively, and those of CVT-10216 at R^1^ and R^2^ position are S2- and -NHSO_2_CH_3_, respectively. **(A)** shows the functional groups of isoflavone analogues addressed in the paper by Ferreira and Fraga (2016), and **(B)** shows the functional groups addressed in the paper by Zhang et al. (2022).

In 2016, Ferreira and Fraga performed molecular docking calculations for 11 isoflavones to construct a pharmacophore model (Ferreira and Fraga, 2016). Daidzin and its analogues, which have been reported as selective inhibitors of ALDH2, were selected for their docking study (Figure 2A). The predicted docked conformation of daidzin to ALDH2 was extremely similar to the X-ray crystal structure (PDB ID: 2VLE), substantiating the reliability of the docking method. Based on the docked conformations of daidzin and its analogues, they built a pharmacophore model consisting of four pharmacophoric points and identified critical interactions in the binding of the inhibitors to ALDH2. In addition to this pharmacophore model, they also found that the outstanding potency of CVT-10216, a highly selective inhibitor of ALDH-2, is attributed to a π-stacking interaction between its aromatic ring in positions R^1^ and Phe292, which is not available in the other inhibitors.

The same group also investigated the molecular basis of the preferred binding of diadzin to ALDH2 over its isoform, ALDH1, using docking and molecular dynamics (MD) simulations (da Silva Cunha et al., 2020). Selective inhibition of ALDH2 is important because simultaneous inhibition of ALDH1 could result in fetal malformation, inflammatory reactions, and vision changes. Based on their docking and MD simulation results, they concluded that the preferred binding of daidzin to ALDH2 over ALDH1 originated from relatively stronger and more stable interactions between daidzin and ALDH2 because ALDH1 has a larger active site cavity, thereby providing more conformational freedom to daidzin when it binds to ALDH1.

Recently, Zhang *et al.* examined the binding pose and strengths of eight isoflavone analogues (Figure 2B), including daidzin and CVT-10216, using molecular docking, MD simulations, and various free energy calculation methods such as molecular mechanics Poisson-Boltzmann surface area (MM-PBSA) analysis, steered MD, and umbrella sampling (Zhang et al., 2022). Based on the binding energy decomposition from the MM-PBSA analysis, they identified the residues that are crucial for the interactions between inhibitors and ALDH2. Moreover, they demonstrated that all the free energy calculation methods employed could successfully reproduce the order of the relative binding strength of inhibitors against ALDH2 obtained from the experimentally determined half-maximal inhibitory concentration. They also pointed out that appropriate consideration of the desolvation of binding partners is critical for the accurate prediction of relative binding strength order, which implies that the desolvation effect is an important factor to be considered when designing new drugs against ALDH2.

All the studies addressed above computationally investigated the interactions between inhibitors and ALDH2 and identified key factors contributing to the interactions, thereby providing valuable clues for designing new drugs against ALDH2. These are good examples showing the role of computer simulations in this field. Such simulations may provide valuable information in a relatively short time and at a low cost, which were only previously obtainable from expensive and time-consuming experiments.

## 4 Conclusions and perspectives

Although the involvement of isoflavone analogues in the progression of ALD has been previously established, the mechanisms underlying the pathophysiology of ALD associated with it remain unclear. Therefore, studying the role of isoflavone analogues and ALDH2 in the pathophysiological pathways of ALD is essential. This mini-review focused on current research in animal models and cells (mainly hepatocytes, macrophages, and other immune cells) on isoflavone analogues reported in ALD. Furthermore, we discussed the capability of computational approaches to inhibit ALDH2 with isoflavone analogues. Emerging studies have established that isoflavone analogues inhibit ALDH2 activity and decrease oxidative stress, inflammation, steatosis, fibrosis, and apoptosis in the liver, eventually protecting against the progression of ALD. Therefore, isoflavone analogues are promising therapeutic candidates for ALD. In the future, further clinical investigation of the relationship between isoflavone analogues and clinical trials will support the development of effective treatments for ALD.

## Abbreviations

ALD, alcoholic liver disease; ALDH2, aldehyde dehydrogenase 2; α-SMA, alpha-smooth muscle actin; ALT, alanine aminotransferase; ASH, alcoholic steatohepatitis; AST, aspartate aminotransferase; CYP2E1, cytochrome P450 2E1; GSK-3β, glycogen synthase kinase-3 beta; HCC, hepatocellular carcinoma; HSC, hepatic stellate cells; IL-6, interleukin-6; LPS, lipopolysaccharide; MAPK, mitogen-activated protein kinase; MCP-1, monocyte chemoattractant protein-1; MD, molecular dynamics; MM-PBSA, molecular mechanics poisson-boltzmann surface area; MPO, myeloperoxidase; NF-κB, nuclear factor-κB; TGF-β1, transforming growth factor-β1; TNBS, trinitrobenzenesulfonic acid; TNF-α, tumor necrosis factor-α.
